# Validation and Performance of Five Upper Gastrointestinal Bleeding Prognostication Scores in a Single-Centre United Kingdom Cohort of over 1000 Patients

**DOI:** 10.3390/jcm15197503

**Published:** 2026-09-27

**Authors:** Mohamed Jalloh, Balamrit Singh Sokhal, Maryam Nusrat Javed, Humberto Gomez, Jessica Lightfoot, Hareesha Rishab Bharadwaj, Arkadeep Dhali, Farhan Gohar

**Affiliations:** 1 Department of Gastroenterology, Royal Stoke University Hospital, University Hospitals of North Midlands NHS Trust, Stoke-on-Trent ST4 6QG, UK; 2 School of Medicine, Keele University, Keele ST5 5BG, UK; 3 Academic Unit of Gastroenterology, Sheffield Teaching Hospitals NHS Foundation Trust, Sheffield S5 7AU, UK; 4 School of medicine, Dentistry and Biomedical Sciences, Queen’s University Belfast, Belfast BT9 7BL, UK

**Keywords:** upper gastrointestinal bleeding, risk stratification, clinical scoring systems, inpatient mortality, inpatient complications, outcomes

## Abstract

**Objectives:** Upper gastrointestinal bleeding (UGIB) is a common medical emergency associated with substantial morbidity and mortality. Several clinical risk scores are available, but their comparative performance across outcomes and patient subgroups remains uncertain. We compared five established UGIB risk scores for predicting inpatient mortality and complications in a large United Kingdom (UK) cohort and evaluated performance according to bleeding aetiology and sex. We also explored the incremental prognostic value of body mass index (BMI). **Methods:** We conducted a retrospective cohort study of 1003 hospitalised patients with UGIB who underwent oesophagogastroduodenoscopy at a UK tertiary centre (2023–2025). Five risk scores were evaluated: age, blood tests, and comorbidities (ABC); mental status–anaesthesiologist score–pulse–albumin–systolic blood pressure–haemoglobin (MAP(ASH)), Charlson comorbidity index–hospital-onset–albumin–mental status–Eastern Cooperative Oncology Group performance status–steroids (CHAMPS), Glasgow–Blatchford score (GBS), and the Japanese score. The primary outcome was inpatient mortality, and the secondary outcome was an inpatient complication composite comprising rebleeding, repeat endoscopy, or interventional radiology. Discrimination was assessed using area under the receiver operating characteristic curves (AUROCs), with pairwise comparisons using the DeLong test. BMI was evaluated using multivariable regression and AUROC analysis. **Results:** ABC demonstrated the highest numerical discrimination for inpatient mortality (AUROC 0.817), followed by CHAMPS (0.791) and MAP(ASH) (0.774), with no statistically significant differences between these three scores. For inpatient complications, MAP(ASH) had the highest numerical AUROC (0.697), although its performance did not differ significantly from CHAMPS, GBS, or ABC. Exploratory subgroup analyses demonstrated generally good mortality discrimination for ABC and CHAMPS across bleeding aetiology and sex. Adding BMI to ABC increased the AUROC from 0.817 to 0.828, although this was not statistically significant (*p* = 0.224). **Conclusions:** ABC, CHAMPS, and MAP(ASH) demonstrated the strongest discrimination for inpatient mortality, while discrimination for inpatient complications was more modest across scores. These findings do not establish the clinical superiority of a single scoring system, and BMI did not provide significant incremental prognostic value.

## 1. Introduction

Upper gastrointestinal bleeding (UGIB) is a common emergency presentation which carries a high risk of morbidity and mortality. The incidence of UGIB is estimated to be between 15 and 172 per 100,000 person-years [1]. Several clinical prediction scores have been developed for risk stratification to support clinical decision-making and guide management [2].

- **Glasgow-Blatchford Score (GBS):** A pre-endoscopy risk score developed primarily to predict the need for clinical intervention following UGIB [3].
- **Age, blood tests, and comorbidities (ABC) score:** Developed to predict mortality in patients presenting with gastrointestinal bleeding [4].
- **Charlson comorbidity Index ≥ 2, in-hospital onset, albumin <2.5 g/dL, altered mental status, Eastern Cooperative Oncology Group performance status ≥2, steroid use score (CHAMPS):** Developed to predict in-hospital mortality in patients with non-variceal UGIB [5].
- **Mental status–anaesthesiologist score–pulse–albumin–systolic blood pressure–haemoglobin (MAP(ASH)) score:** Developed to predict intervention and mortality in patients with UGIB [6].
- **Japanese score:** Developed to predict the need for therapeutic intervention in patients with UGIB [7].

Although these risk scores were developed for different clinical purposes and populations, including prediction of clinical intervention, mortality, or both, they may provide complementary information when assessing patients presenting with UGIB. Their performance against a common outcome should therefore be interpreted in the context of their original intended purpose, rather than as evidence that one score is globally superior to another. Direct comparison across clinically relevant outcomes may nevertheless provide useful information regarding their relative prognostic performance.

Despite the development of several UGIB risk scores, comparative validation studies from the United Kingdom (UK) remain limited, particularly for newer scores such as ABC, CHAMPS, and MAP(ASH). GBS remains widely used in clinical practice, while newer scoring systems have undergone comparatively less validation across different populations and outcomes. Further evaluation within contemporary UK cohorts may therefore help determine how their performance compares within local clinical practice.

An additional underexplored factor is body mass index (BMI). BMI may reflect aspects of nutritional status and body composition that are not directly incorporated into established UGIB risk scores, although its prognostic value in UGIB remains uncertain. Evaluation of BMI may therefore provide exploratory information regarding whether it offers additional prognostic value beyond established risk stratification tools.

In this large single-centre cohort from a UK tertiary hospital, we aimed to compare the performance of five UGIB risk scores for predicting all-cause inpatient mortality and a study-specific inpatient complication composite. We additionally evaluated score performance in exploratory subgroups according to bleeding aetiology and sex, assessed liver disease severity among patients with cirrhosis, and explored the incremental prognostic value of BMI.

## 2. Methods

### 2.1. Ethical Approval

This project was reviewed and registered by the Royal Stoke University Hospital Audit Department as a clinical audit/service evaluation (Clinical Audit ID CA09425). Local governance review determined that formal Research Ethics Committee approval was not required. As this study involved the retrospective analysis of anonymised routinely collected clinical data, individual patient consent was not required in accordance with local governance policies.

### 2.2. Study Design, Population and Setting

This retrospective cohort study was conducted between August 2023 and January 2025. Consecutive patients admitted to a UK tertiary referral centre with suspected UGIB who underwent oesophagogastroduodenoscopy (OGD) were included. The source cohort comprised patients who underwent inpatient OGD; patients with suspected UGIB who did not undergo inpatient endoscopy were therefore not captured within the study dataset.

### 2.3. Data Collection

Clinical data were retrospectively extracted from the hospital’s electronic medical records. Variables collected included presenting symptoms, clinical signs, admission vital signs, Eastern Cooperative Oncology Group performance status (ECOG-PS), American Society of Anaesthesiologists (ASA) score, laboratory markers, medication history, comorbidities, and patient characteristics. Further details are provided in the Supplementary Methods.

Ethnicity was extracted from routinely recorded electronic health record data using the categories available in the source record (Caucasian, African, Asian, Mixed, or not recorded).

BMI was categorised according to World Health Organization (WHO) criteria as underweight (<18.5), healthy weight (18.5–24.9), overweight (25.0–29.9), obese (30.0–39.9), and severely obese (≥40) [8].

The collected data were used to retrospectively reconstruct five established UGIB risk scores: GBS (0–23), ABC (0–18), MAP(ASH) (0–9), CHAMPS (0–7), and the Japanese score (−4 to 10), according to their original published definitions and point allocations, without refitting score weights [3,4,5,6,7]. Laboratory components were derived from the first blood tests obtained on admission, while physiological variables, including systolic blood pressure and heart rate, were obtained from the first recorded observations on admission. Clinical variables and comorbidities were extracted retrospectively from the electronic health record. ASA physical status, ECOG performance status, and altered mental status were determined from the documented clinical information available in the electronic record. The component definitions and data sources used to reconstruct each score are provided in Supplementary Table S1.

Score reconstruction was based on the established component definitions and thresholds and was not modified according to observed outcomes. As this was a retrospective electronic-record study, outcome information was contained within the same clinical record and formal outcome blinding was not feasible.

These five scores were selected because they comprise both established (GBS) and newer (ABC, MAP(ASH), CHAMPS, and Japanese) UGIB-specific risk scores with differing intended clinical applications, including prediction of mortality, clinical intervention, or both. Their performance was therefore compared against the common study outcomes rather than interpreted as a comparison of their original intended purposes. Other established scores, including Rockall and AIMS65 (albumin, international normalized ratio, altered mental status, systolic blood pressure, and age ≥65 years), were not evaluated, as comparison of every available UGIB prognostic score was beyond the scope of the study.

### 2.4. Outcomes

The primary outcome was all-cause inpatient mortality during the index admission. The secondary outcome was an inpatient complication composite, defined as rebleeding, requirement for repeat OGD, or requirement for an interventional radiology procedure. These components were not mutually exclusive. Rebleeding was defined as recurrent upper gastrointestinal bleeding, evidenced by haematemesis, melaena, or coffee-ground vomiting. Transfusion, index endoscopic haemostasis, surgery, and intensive care admission were not independently encoded components of the study-specific secondary endpoint. The predictive performance of each risk score was evaluated across both outcomes.

### 2.5. Statistical Analysis

Normally distributed continuous variables are presented as mean ± standard deviation (SD), while non-normally distributed variables are reported as median and interquartile range (IQR). Categorical variables are presented as frequencies and percentages.

Comparisons between variceal and non-variceal bleeding were performed using the independent samples *t*-test for normally distributed variables, the Mann–Whitney U test for non-normally distributed variables, and the chi-square or Fisher’s exact test for categorical variables, as appropriate. Statistical significance was defined as *p* < 0.05.

The performance of evaluated low-risk thresholds for inpatient mortality was assessed using ABC ≤ 3, CHAMPS = 0, GBS ≤ 1, MAP(ASH) ≤ 2, and Japanese score ≤ 0 [6,9,10,11]. Sensitivity, specificity, positive predictive value (PPV), and negative predictive value (NPV), with corresponding 95% confidence intervals, were calculated for each threshold.

Discriminatory performance was assessed using area under the receiver operating characteristic curves (AUROCs) with corresponding 95% confidence intervals (95% CI) and *p*-values. AUROCs were compared using the DeLong test. The relationship between each score and inpatient mortality was additionally illustrated using observed mortality at each score value and mortality probabilities estimated from logistic regression models fitted within the study cohort. These locally fitted models were used descriptively and were not considered an independent assessment of the absolute-risk calibration of the original scoring systems.

Exploratory subgroup analyses evaluated score performance according to bleeding aetiology (variceal versus non-variceal) and sex. Formal effect modification was assessed using logistic regression models incorporating multiplicative interaction terms (e.g., ABC × sex), with significance evaluated using Wald tests (*p* < 0.05). Given the smaller number of patients and events within some subgroups, these analyses were considered exploratory.

Additional analyses were performed among patients with documented cirrhosis.International normalized ratio (INR), platelet count, and Model for End-Stage Liver Disease–sodium (MELD-Na)were compared between patients who survived and those who died during the index admission. Within the variceal bleeding subgroup, the discriminatory performance of ABC was additionally compared with MELD-Na for inpatient mortality using the DeLong test.

Additional exploratory analyses evaluated the role of BMI. Multivariable logistic regression adjusted for the ABC score was performed among patients with recorded BMI to assess the association between BMI category and inpatient mortality. Patients with and without recorded BMI were also compared according to age, sex, ABC score, bleeding aetiology, and inpatient mortality to assess potential differences associated with BMI missingness. Finally, BMI was incorporated into a model containing the ABC score, and the change in discriminatory performance for inpatient mortality was assessed using AUROC analysis.

Missing data were handled using analysis-specific complete-case analyses. The overall cohort comprised 1003 patients, with participants contributing to all analyses for which complete data were available and being excluded only from analyses requiring missing variables. Missing data primarily affected secondary analyses involving BMI and MELD-Na, while missingness for variables used in the primary analyses was minimal. The extent of missing data for variables included in the analyses is reported in Supplementary Table S2.

All statistical analyses were performed using IBM SPSS Statistics version 29.0 and R version 4.5.0.

## 3. Results

### 3.1. Baseline Characteristics

A total of 1003 patients who underwent inpatient OGD during the study period were included (Figure 1). Baseline characteristics are summarised in Table 1. The median age was 71 years, and 594 (59.2%) patients were male. Overall, 921 (91.8%) patients were recorded as Caucasian, 17 (1.7%) as Asian, 12 (1.2%) as Mixed, 8 (0.8%) as African, and ethnicity was not recorded in 45 (4.5%) patients. The most prevalent comorbidities were hypertension (47.1%), diabetes (27.6%), lung disease (22.1%), active cancer (19.9%), and liver cirrhosis (17.8%). BMI data were available for 748 (74.6%) patients, with a median BMI of 26.2 kg/m^2^ (IQR 23–30).

Baseline characteristics stratified by bleeding aetiology are presented in Supplementary Table S3. Patients with variceal bleeding (*n* = 107) were younger than those with non-variceal bleeding (*n* = 896) (median age 61 [IQR 55–67] vs. 73 [IQR 60–82] years; *p* < 0.001). Patients with variceal bleeding also had higher MELD-Na scores (16 [IQR 12–20.75] vs. 9.5 [IQR 8–14]; *p* < 0.001) and higher rates of decompensated cirrhosis (76.6% vs. 7.8%; *p* < 0.001).

### 3.2. Crude Outcomes and Complications

Overall, 94 (9.4%) patients died during the index admission, and 77 (7.7%) experienced the inpatient complication composite. Among patients experiencing the composite outcome, 42 experienced rebleeding, 46 required repeat OGD, and 10 required an interventional radiology procedure. These components were not mutually exclusive: 18 patients experienced both rebleeding and repeat OGD, one experienced rebleeding and interventional radiology, and two experienced repeat OGD and interventional radiology; no patient experienced all three components.

Gastrointestinal causes accounted for the largest proportion of inpatient deaths (21, 22.3%), followed by respiratory (11, 11.7%) and cardiovascular causes (10, 10.6%) (Table 1). Patients with variceal bleeding experienced significantly higher rates of the inpatient complication composite than those with non-variceal bleeding (15.9% vs. 6.7%; *p* < 0.001). However, all-cause inpatient mortality did not differ significantly between bleeding aetiologies (14.0% vs. 8.8%; *p* = 0.081) (Supplementary Table S3).

Using the extracted clinical data, ABC, GBS, CHAMPS, MAP(ASH), and Japanese scores were reconstructed for each patient with the required available data, with their median (IQR) values presented in Table 1.

### 3.3. Comparison of UGIB Risk Scores

#### 3.3.1. Inpatient Mortality

AUROC analysis for inpatient mortality showed that ABC had the numerically highest discrimination, with an AUROC of 0.817 (95% CI: 0.779–0.855), followed by CHAMPS (AUROC: 0.791, 95% CI: 0.745–0.836), MAP(ASH) (AUROC: 0.774, 95% CI: 0.731–0.816), and GBS (AUROC: 0.713, 95% CI: 0.660–0.765). The Japanese score had the lowest discrimination, with an AUROC of 0.613 (95% CI: 0.553–0.674) (Table 2; Figure 2).

Pairwise DeLong comparisons demonstrated no statistically significant differences between ABC and CHAMPS (ΔAUROC 0.026, 95% CI −0.015 to 0.067; *p* = 0.221), ABC and MAP(ASH) (ΔAUROC 0.043, 95% CI −0.004 to 0.090; *p* = 0.070), or CHAMPS and MAP(ASH) (ΔAUROC 0.017, 95% CI −0.025 to 0.059; *p* = 0.421). ABC, CHAMPS, and MAP(ASH) each demonstrated significantly greater discrimination than GBS, while the Japanese score demonstrated significantly lower discrimination than each of the other four scores (Supplementary Table S4).

Observed inpatient mortality generally increased with increasing values of the five risk scores. The observed and locally fitted score-to-mortality relationships are presented in Figure 3.

At the evaluated low-risk thresholds, ABC ≤3 classified 383/1002 (38.2%) patients as low risk, with a sensitivity of 96.8% and NPV of 99.2%. CHAMPS = 0 classified 91/999 (9.1%) patients as low risk, with a sensitivity of 98.9% and NPV of 98.9%, while MAP(ASH) ≤2 classified 492/999 (49.2%) patients as low risk, with a sensitivity of 89.4% and NPV of 98.0%. Corresponding sensitivity, specificity, PPV, and NPV estimates with 95% confidence intervals for all five scores are presented in Supplementary Table S5.

#### 3.3.2. Inpatient Complications

For the inpatient complication composite, MAP(ASH) had the numerically highest AUROC at 0.697 (95% CI: 0.645–0.748), followed by CHAMPS (AUROC: 0.671, 95% CI: 0.612–0.730), GBS (AUROC: 0.670, 95% CI: 0.613–0.727), and ABC (AUROC: 0.651, 95% CI: 0.592–0.709). The Japanese score demonstrated the lowest discrimination (AUROC: 0.550, 95% CI: 0.479–0.621) (Table 2; Figure 2).

Pairwise comparisons demonstrated no statistically significant differences among ABC, MAP(ASH), CHAMPS, and GBS for predicting the inpatient complication composite. The Japanese score demonstrated significantly lower discrimination than each of the other four scores (Supplementary Table S4).

#### 3.3.3. Exploratory Subgroup Analyses by Bleeding Aetiology and Sex

In subgroup analyses according to bleeding aetiology, ABC demonstrated an AUROC of 0.802 (95% CI: 0.674–0.930) in patients with variceal bleeding and 0.818 (95% CI: 0.777–0.859) in those with non-variceal bleeding. CHAMPS demonstrated AUROCs of 0.743 and 0.799 in the variceal and non-variceal groups, respectively, while corresponding MAP(ASH) AUROCs were 0.610 and 0.789. GBS and the Japanese score demonstrated lower discrimination in the variceal subgroup, with AUROCs of 0.565 and 0.584, respectively (Table 2; Supplementary Figure S1).

Within the variceal subgroup, ABC was additionally compared with MELD-Na for predicting inpatient mortality. Among the 106 patients with complete data for both scores, including 15 inpatient deaths, ABC demonstrated an AUROC of 0.800 (95% CI 0.672–0.929), compared with 0.601 (95% CI 0.442–0.761) for MELD-Na. The difference in AUROC was 0.199 (95% CI 0.002–0.396; *p* = 0.048) (Supplementary Table S6).

For inpatient mortality, ABC demonstrated AUROCs of 0.805 in males and 0.836 in females. Corresponding AUROCs were 0.788 and 0.793 for CHAMPS, 0.767 and 0.781 for MAP(ASH), and 0.691 and 0.743 for GBS. The Japanese score demonstrated lower discrimination in both groups (Table 2; Supplementary Figure S1). No statistically significant interactions between sex and the evaluated scores were observed (all *p* > 0.05). Given the smaller numbers of patients and events within some subgroups, these analyses were considered exploratory.

Supplementary Figure S2 illustrates the comparative AUROCs of the five scores for inpatient mortality across bleeding-aetiology and sex subgroups.

#### 3.3.4. Additional Analyses Among Patients with Cirrhosis

Among 178 patients with documented cirrhosis, 149 survived, and 29 died during the index admission. Patients who died had a higher median MELD-Na score than those who survived (21 [IQR 15.8–23] vs. 15 [IQR 11.2–20]; *p* = 0.002). Median INR was numerically higher among patients who died (1.65 [IQR 1.58–1.90] vs. 1.50 [IQR 1.30–1.87]; *p* = 0.057), while median platelet count was numerically lower (124 [IQR 84–172] vs. 155 [IQR 86–205] ×10^9^/L; *p* = 0.307); neither difference was statistically significant (Supplementary Table S7). Inpatient mortality occurred in 28/152 (18.4%) patients with decompensated cirrhosis compared with 1/26 (3.8%) patients with compensated cirrhosis (Fisher’s exact *p* = 0.083).

#### 3.3.5. Additional Exploratory Analyses of BMI

Among the 748 patients with recorded BMI, multivariable logistic regression adjusted for ABC score demonstrated no statistically significant overall association between BMI category and all-cause inpatient mortality (likelihood-ratio *p* = 0.702). Compared with patients of healthy weight, no individual BMI category was significantly associated with inpatient mortality. ABC remained independently associated with mortality (adjusted OR 1.459, 95% CI 1.327–1.616; *p* < 0.001) (Supplementary Table S8).

Patients with and without recorded BMI differed in several characteristics. Patients with recorded BMI were older (68.2 ± 16.1 vs. 62.1 ± 20.4 years; *p* < 0.001) and had a higher mean ABC score (4.87 ± 3.04 vs. 4.25 ± 3.35; *p* = 0.010). Inpatient mortality was lower among patients with recorded BMI than among those without recorded BMI (7.9% vs. 13.7%; *p* = 0.009). There were no statistically significant differences in sex or variceal bleeding status between the groups (Supplementary Table S9).

Incorporation of BMI into the ABC model resulted in a modest numerical increase in discrimination for inpatient mortality, from an AUROC of 0.817 to 0.828; however, this difference was not statistically significant (*p* = 0.224) (Supplementary Figure S3; Supplementary Table S10).

## 4. Discussion

### 4.1. Summary of Findings

This is the first large single-centre cohort study from a UK tertiary hospital directly comparing five UGIB risk scores. Our findings demonstrate that ABC, CHAMPS, and MAP(ASH) had the strongest discrimination for all-cause inpatient mortality, with ABC achieving the numerically highest AUROC. However, pairwise comparisons demonstrated no statistically significant differences between these three scores. For inpatient complications, MAP(ASH) achieved the highest numerical AUROC, although its performance did not differ significantly from CHAMPS, GBS, or ABC. These findings demonstrate differences in prognostic performance but do not establish the clinical superiority of any single scoring system.

Exploratory subgroup analyses demonstrated generally good mortality discrimination for ABC and CHAMPS across bleeding aetiology and sex, although estimates were less precise in smaller subgroups, particularly patients with variceal bleeding. Low-risk threshold analyses demonstrated high negative predictive values for several scores, but the proportion of patients classified as low risk varied substantially between thresholds. Finally, exploratory BMI analyses did not demonstrate a statistically significant independent association between BMI category and inpatient mortality after adjustment for ABC among patients with recorded BMI. Incorporation of BMI into the ABC model produced only a modest, non-significant improvement in discrimination.

Upper gastrointestinal bleeding (UGIB) remains a common medical emergency with an inpatient mortality of approximately 10%, emphasising the importance of accurate early risk stratification [4]. Although several UGIB risk scores have been developed, GBS remains widely adopted in routine clinical practice, while newer scores have undergone comparatively less external validation across different outcomes and patient populations. Our study therefore provides additional real-world evidence regarding the comparative performance of contemporary UGIB risk scores within a UK tertiary hospital setting.

### 4.2. Validation of ABC, MAP(ASH) and CHAMPS Scores as Predictors of Inpatient Mortality

In our cohort, ABC, MAP(ASH), and CHAMPS demonstrated the strongest discrimination for inpatient mortality, consistent with external validation studies across diverse populations [11,12,13]. Importantly, although ABC achieved the numerically highest AUROC, pairwise comparisons demonstrated no statistically significant differences between ABC, CHAMPS, and MAP(ASH). ABC, CHAMPS, and MAP(ASH) did, however, demonstrate significantly greater discrimination than GBS, while the Japanese score showed the lowest discrimination.

The performance of these scores may partly reflect their incorporation of markers of systemic illness and comorbidity in addition to variables related to acute bleeding. MAP(ASH) incorporates albumin, haemoglobin, ASA score, and mental status, whereas CHAMPS incorporates factors including ECOG performance status, albumin, mental status, and comorbidity burden [5,6]. ABC similarly incorporates age, biochemical markers, ASA status, and important comorbidities [4]. These factors may be particularly relevant in hospitalised patients, where mortality may reflect both the acute bleeding episode and underlying systemic illness.

In addition to identifying patients at increased risk of mortality, several evaluated low-risk thresholds demonstrated high negative predictive values. However, the proportion classified as low risk varied substantially between scores. ABC ≤ 3 classified 38.2% of evaluable patients as low risk with an NPV of 99.2%, whereas CHAMPS = 0 classified 9.1% as low risk with an NPV of 98.9%. MAP(ASH) ≤ 2 classified 49.2% as low risk with an NPV of 98.0%. These findings demonstrate differences in the operating characteristics of individual thresholds and should not be interpreted as establishing their safety for discharge, particularly as the present cohort was restricted to hospitalised patients undergoing inpatient endoscopy [9,10,11,12].

### 4.3. UGIB Risk Scores for Predicting Inpatient Complications

In our cohort, MAP(ASH), CHAMPS, and GBS demonstrated the highest numerical AUROCs for predicting the inpatient complication composite. This is consistent with previous studies identifying MAP(ASH) as predictive of intervention and rebleeding, GBS as a predictor of intervention and endoscopic therapy, and CHAMPS as a potential predictor of adverse outcomes [6,10,11,14].

These scores incorporate acute physiological variables relevant to UGIB-related deterioration. GBS reflects acute bleeding severity through variables including haemoglobin, urea, blood pressure, and heart rate, whereas MAP(ASH) and CHAMPS additionally incorporate markers of systemic illness and physiological reserve [10,14]. However, pairwise comparisons in the present cohort demonstrated no statistically significant differences between ABC, MAP(ASH), CHAMPS, and GBS for the complication composite. The comparatively modest AUROCs across all scores also suggest that prediction of subsequent inpatient complications may be more difficult than prediction of mortality.

The secondary outcome should additionally be considered in the context of its composite definition. Rebleeding, repeat OGD, and interventional radiology were not mutually exclusive, and repeat OGD may occur as a consequence of rebleeding. Furthermore, the individual components occurred less frequently than the composite outcome, particularly interventional radiology. These factors should be considered when interpreting the discriminatory performance observed for this endpoint.

### 4.4. Validation of UGIB Risk Score Performance Across Bleeding Aetiology and Sex Subgroups

Exploratory subgroup analyses demonstrated generally good mortality discrimination for ABC and CHAMPS across bleeding aetiology and sex. MAP(ASH) demonstrated lower numerical discrimination in variceal compared with non-variceal bleeding, although formal interaction analysis did not demonstrate statistically significant effect modification by bleeding aetiology. This pattern is consistent with previous studies reporting good mortality discrimination for ABC, CHAMPS, and MAP(ASH) [5,12,15,16]. More broadly, a contemporary management review emphasises the role of risk stratification in guiding the assessment and management of patients presenting with UGIB [17]. However, the smaller sample size and number of deaths within the variceal subgroup resulted in wider confidence intervals, and the absence of a statistically significant interaction should not be interpreted as evidence of equivalent performance between subgroups.

The lower numerical performance of MAP(ASH) in variceal bleeding has also been reported previously. Jimenez-Rosales et al. [11] suggested that variables such as systolic blood pressure, heart rate, and haemoglobin may be affected by the physiological consequences of chronic liver disease and portal hypertension, potentially altering their relationship with acute bleeding severity. Our subgroup findings should nevertheless be regarded as exploratory and require confirmation in larger cohorts.

Within patients with variceal bleeding, ABC demonstrated greater discrimination for inpatient mortality than MELD-Na (AUROC 0.800 versus 0.601; *p* = 0.048). This suggests that a UGIB-specific score incorporating acute clinical features and broader systemic factors may capture prognostic information not represented by a liver disease severity score alone. However, this analysis included only 106 patients and 15 inpatient deaths and should therefore be interpreted cautiously.

Among patients with documented cirrhosis, those who died during admission had significantly higher MELD-Na scores than survivors. INR was numerically higher, and platelet count numerically lower among those who died, although neither difference reached statistical significance. These findings highlight the heterogeneity of patients with cirrhosis presenting with UGIB and the potential importance of underlying liver disease severity when interpreting general UGIB risk scores.

Importantly, variceal bleeding is not synonymous with cirrhosis. Portal hypertension and variceal bleeding may occur in non-cirrhotic conditions. Recent literature has highlighted the heterogeneity of primary biliary cholangitis (PBC), including portal-hypertensive disease, while porto-sinusoidal vascular disease (PSVD) represents an important cause of portal hypertension in the absence of cirrhosis [18,19]. The five UGIB scores evaluated in the present study have not been specifically validated in these individual patient groups. Consequently, their performance in patients with non-cirrhotic portal hypertension remains uncertain and warrants further evaluation.

### 4.5. Clinical Interpretation of Score Performance

The present findings support differences in prognostic discrimination between the evaluated UGIB scores but do not establish the clinical superiority of a single scoring system. ABC achieved the highest numerical AUROC for inpatient mortality, but its performance was not statistically different from CHAMPS or MAP(ASH). Similarly, although MAP(ASH) achieved the highest numerical AUROC for inpatient complications, it was not statistically superior to ABC, CHAMPS, or GBS.

The scores were also developed for differing clinical purposes, and therefore their performance against a common study outcome should not be interpreted as evidence that one score is globally superior to another. Furthermore, discrimination alone does not establish clinical utility or demonstrate that implementation of a score improves patient management or outcomes. Prospective studies evaluating how these scores perform within defined clinical pathways are therefore required before preferential use of a single score can be recommended.

### 4.6. Role of BMI in UGIB Mortality

In the complete-case analysis, BMI category was not significantly associated with inpatient mortality after adjustment for ABC score. Similarly, incorporation of BMI into the ABC model resulted in only a modest numerical increase in discrimination from an AUROC of 0.817 to 0.828, which was not statistically significant. Collectively, these findings do not demonstrate incremental prognostic value from incorporating BMI into ABC.

Interpretation of the BMI analyses is limited by missing data. BMI was unavailable for approximately one-quarter of the cohort, and patients without recorded BMI differed from those with recorded BMI in several characteristics. In particular, patients without recorded BMI were younger and had lower mean ABC scores, but experienced higher inpatient mortality. This raises the possibility of selection bias within the complete-case BMI analysis and limits conclusions regarding the relationship between BMI and inpatient mortality.

BMI also remains a relatively crude measure of body composition and does not distinguish between adipose tissue and muscle mass or account for fat distribution. Consequently, potentially relevant factors such as sarcopenia may not be adequately represented by BMI alone. Given the missingness and lack of a statistically significant incremental improvement in discrimination, the present BMI findings should be considered exploratory and do not support incorporation of BMI into existing UGIB risk scores. Future studies incorporating more direct measures of body composition may provide additional insight into whether nutritional or body-composition measures have prognostic value in UGIB.

### 4.7. Strengths and Limitations

This study has several strengths. It utilises real-world data from a large cohort of patients undergoing inpatient OGD at a UK tertiary centre, capturing the clinical complexity of hospitalised UGIB. The age profile, male predominance, and bleeding patterns showed similarities to those reported in the NCEPOD UGIB audit and the large national study by Crooks et al. [20,21]. However, these similarities do not overcome the limitations inherent to a single-centre cohort. Furthermore, the study directly compared five contemporary UGIB risk scores across mortality and complication outcomes and included exploratory analyses according to bleeding aetiology, sex, liver disease severity, and BMI.

Several limitations should be acknowledged. First, selection of patients through inpatient endoscopy introduces potential spectrum and selection bias. Patients with suspected UGIB who did not undergo inpatient OGD were not represented in the source dataset, and their number and characteristics could not be determined retrospectively. Consequently, the findings are most directly applicable to hospitalised patients with UGIB selected for inpatient endoscopy rather than to all patients presenting with suspected UGIB. The single-centre design further limits generalisability to other settings and healthcare systems.

In addition, the retrospective design is susceptible to incomplete data capture and residual confounding, including unmeasured factors such as treatment decisions and decisions regarding escalation of care. Although the overall cohort was large, some subgroup analyses, particularly those involving patients with variceal bleeding, contained relatively small numbers of patients and outcome events. These analyses should therefore be considered exploratory, and the absence of statistically significant interaction should not be interpreted as evidence of equivalent score performance between subgroups.

Missing data may also have introduced selection bias, particularly in the exploratory BMI analyses. BMI was unavailable for 25.4% of the cohort, and patients without recorded BMI differed from those with recorded BMI in age, ABC score, and inpatient mortality. Although analysis-specific complete-case analyses allowed patients to contribute to analyses for which data were available, these differences limit interpretation of the BMI findings (Supplementary Tables S2 and S9). MELD-Na was unavailable in 7.5% of the overall cohort, and analyses involving liver disease severity should similarly be interpreted in this context. Finally, although the study period occurred after the acute phases of the COVID-19 pandemic, residual effects on healthcare delivery and patient behaviour may have influenced case mix and patterns of hospital presentation.

### 4.8. Clinical Implications and Future Research

The findings of this study demonstrate differences in prognostic discrimination between contemporary UGIB risk scores but do not establish the clinical superiority of a single scoring system. ABC, CHAMPS, and MAP(ASH) demonstrated the strongest discrimination for inpatient mortality, while discrimination for the inpatient complication composite was more modest across scores. The choice of risk score should therefore take into account its intended clinical purpose and the population in which it has been validated rather than relying solely on numerical differences in AUROC.

Practical considerations, including the availability of individual score components and ease of implementation, are also relevant. Some scores require variables such as ASA classification or ECOG performance status that may not be routinely documented at initial assessment. However, the present study evaluated prognostic performance rather than implementation and did not assess whether use of any score improves clinical decision-making or patient outcomes. Prospective studies evaluating score-guided clinical pathways, together with multicentre external validation, are therefore required before preferential use of a single scoring system can be recommended.

Future research should also evaluate these scores in larger variceal and non-cirrhotic portal hypertension populations and assess whether more direct measures of nutritional status or body composition provide incremental prognostic information beyond established UGIB risk scores.

## 5. Conclusions

In conclusion, this study evaluated the performance of five UGIB risk scores in a large single-centre cohort of hospitalised patients undergoing inpatient endoscopy. ABC, CHAMPS, and MAP(ASH) demonstrated the strongest discrimination for inpatient mortality, with ABC achieving the highest numerical AUROC, although differences between these three scores were not statistically significant. Discrimination for the inpatient complication composite was more modest across scores. Exploratory analyses did not demonstrate significant incremental prognostic value from incorporating BMI into ABC.

These findings demonstrate differences in prognostic performance but do not establish the clinical superiority or utility of any single scoring system. Further prospective multicentre evaluation is required to determine whether incorporation of these scores into clinical pathways improves decision-making or patient outcomes.

## Figures and Tables

**Figure 1:**
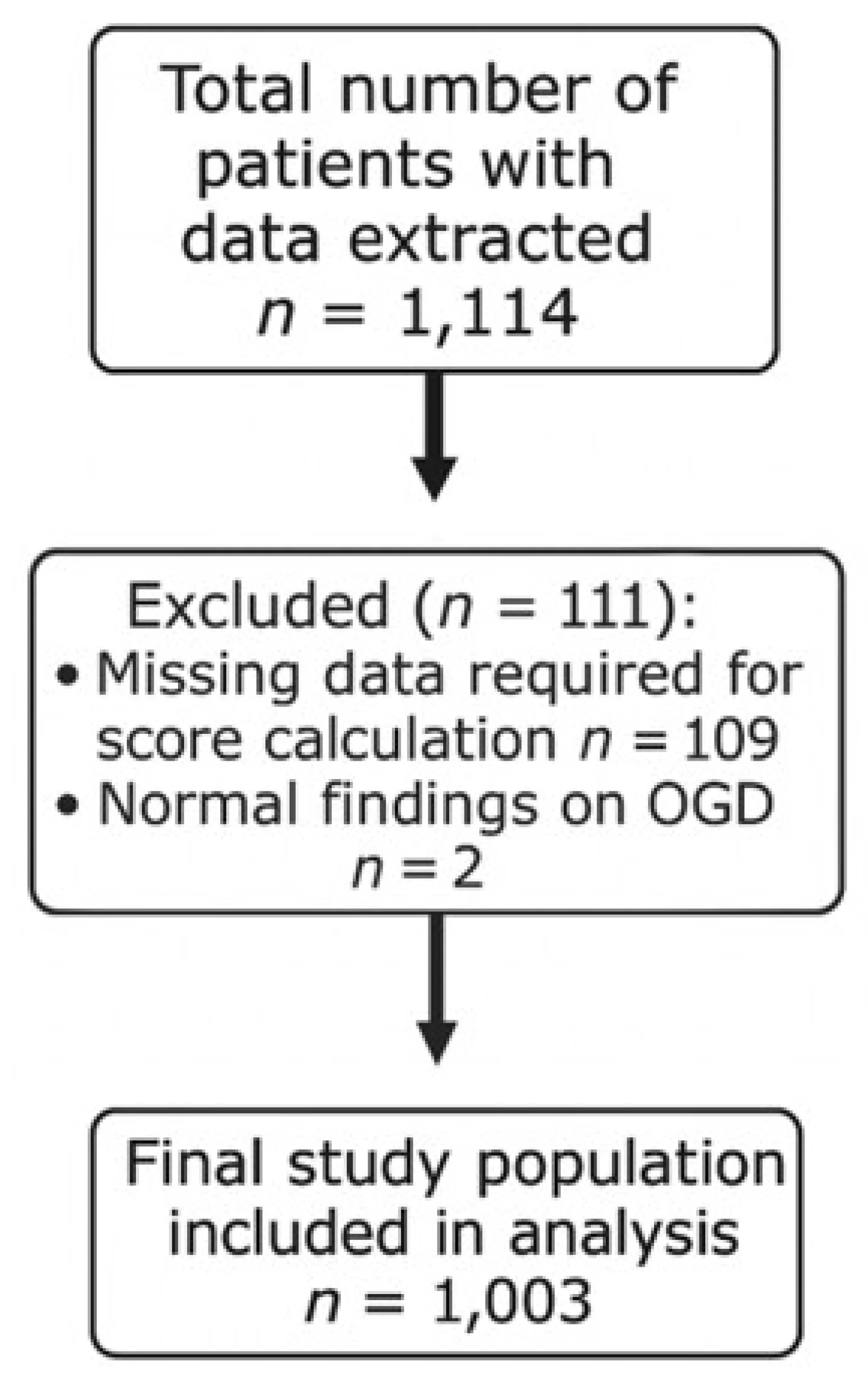
Flowchart of patient inclusion and exclusion criteria. Of 1114 patients with data extracted from the inpatient oesophagogastroduodenoscopy (OGD) cohort, 111 were excluded, leaving 1003 patients in the final study population.

**Figure 2:**
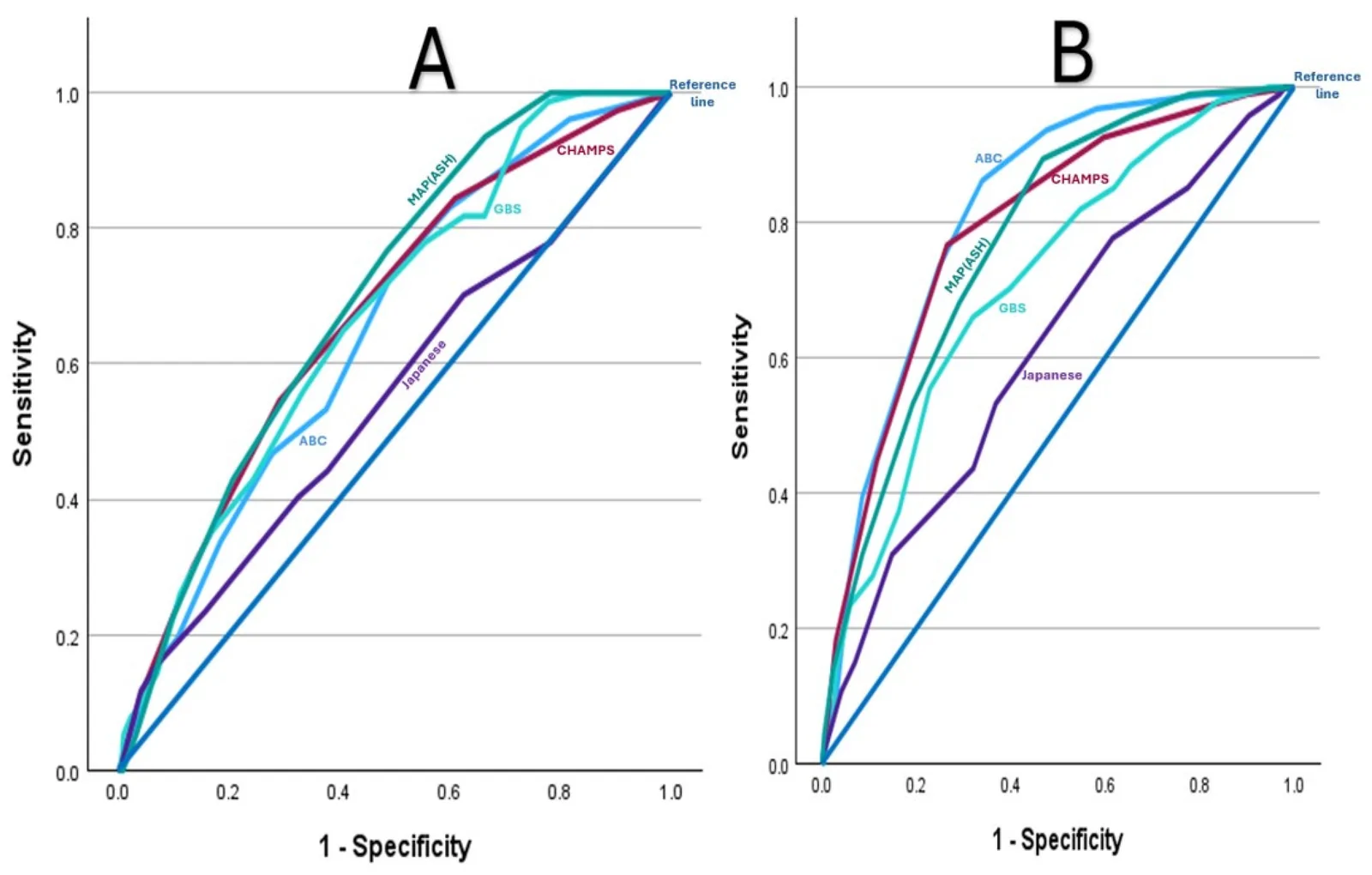
Receiver operating characteristic curves showing the discriminatory performance of the five risk scores in the total cohort for (**A**) the inpatient complication composite and (**B**) all-cause inpatient mortality.

**Figure 3:**
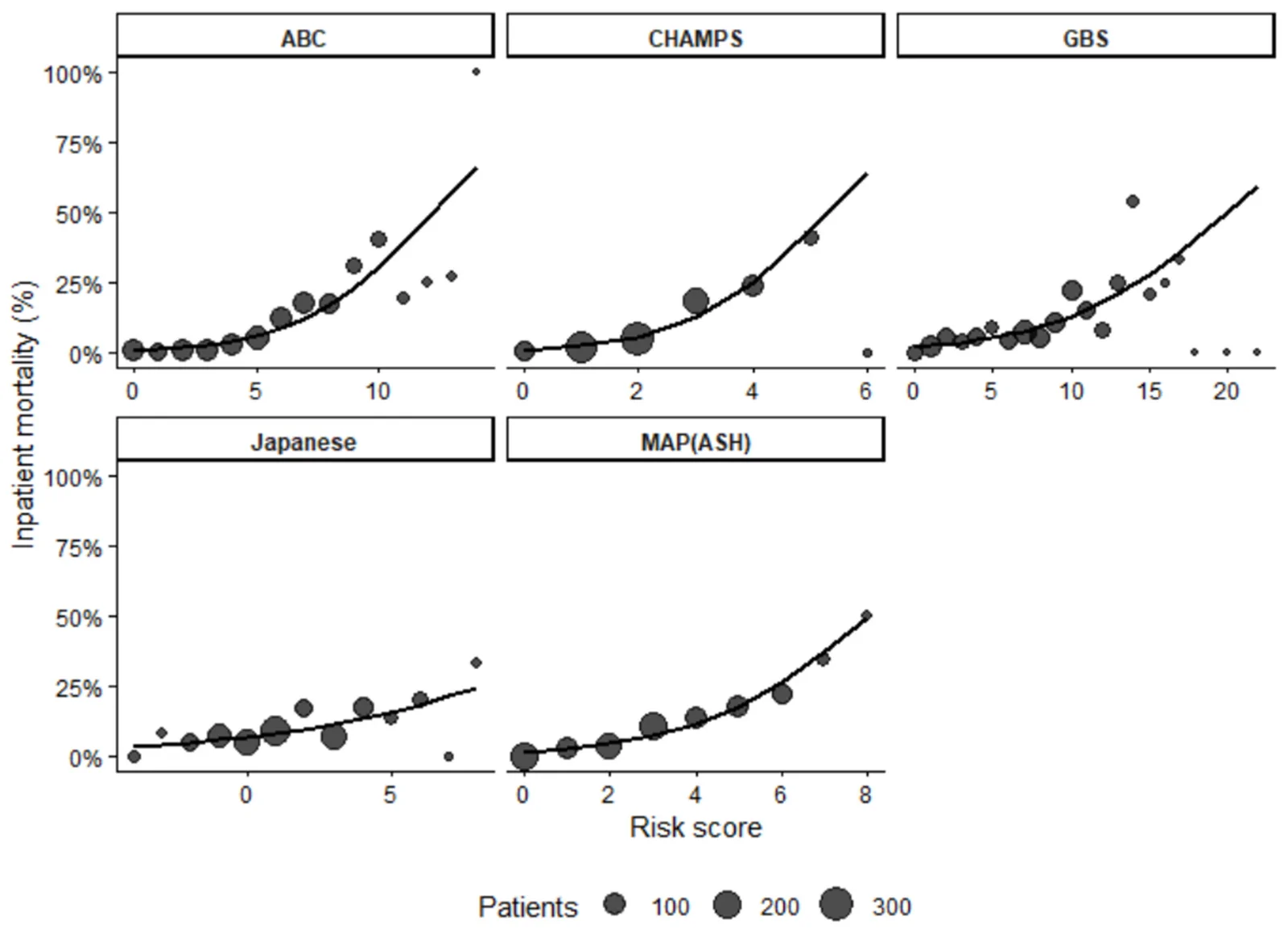
Observed and locally fitted inpatient mortality according to risk score. Points represent observed inpatient mortality at each score value, with point size proportional to the number of patients; lines represent mortality probabilities estimated from logistic models fitted within the study cohort.

**Table 1:** Baseline characteristics of the study population (*n* = 1003), including demographic, clinical, and biochemical data, comorbidities, medications, Risk scores, outcomes, and cause of death.

**Demographic**	
Male, *n* (%)	594 (59.2%)
Age, Median (IQR)	71 (59–81)
**Ethnicity**	
- Caucasian, *n* (%)	921 (91.8%)
- Asian, *n* (%)	17 (1.7%)
- Mixed, *n* (%)	12 (1.2%)
- African, *n* (%)	8 (0.8%)
- Not recorded, *n* (%)	45 (4.5%)
BMI, Median (IQR)	26.2 (23–30)
ECOG: 0–2, *n* (%)	715 (71.3%)
ECOG: 3–4, *n* (%)	288 (28.7%)
**UGIB Aetiology**	
Non-Variceal Bleed, *n* (%)	896 (89.3%)
**Vital Signs on Admission**	
SBP, Median (IQR)	125 (111–140)
HR, Median (IQR)	86 (75–98)
**Laboratory Blood Tests**	
Sodium (mmol/L), Median (IQR)	139 (136–141)
Urea (mmol/L), Median (IQR)	8.4 (5.2–14.0)
Creatinine (µmol/L), Median (IQR)	79 (61–112)
eGFR, Median (IQR)	80 (49–90)
Albumin (g/L), Mean ± SD	28.19 ± 7.76
Haemoglobin (g/L), Median (IQR)	95 (79–117)
**Comorbidities**	
MI, *n* (%)	119 (11.9%)
CCF, *n* (%)	116 (11.6%)
CVA (stroke/TIA), *n* (%)	111 (11.1%)
HTN, *n* (%)	472 (47.1%)
IDA, *n* (%)	83 (8.3%)
Dementia, *n* (%)	46 (4.6%)
Lung Disease (COPD/Asthma/ILD), *n* (%)	222 (22.1%)
Compensated Liver Cirrhosis, *n* (%)	26 (2.6%)
Decompensated Liver Cirrhosis, *n* (%)	152 (15.2%)
Diabetes: Lifestyle controlled, *n* (%)	70 (7.0%)
Diabetes: On 1 medication, *n* (%)	125 (12.5%)
Diabetes: On >1 medication, *n* (%)	81 (8.1%)
CKD Stage 4–5, *n* (%)	62 (6.2%)
Active Cancer: Non-metastatic, *n* (%)	111 (11.1%)
Active Cancer: Metastatic, *n* (%)	89 (8.9%)
**Medications**	
Anticoagulant or Antiplatelet, *n* (%)	398 (39.7%)
NSAIDs, *n* (%)	47 (4.7%)
Steroid, *n* (%)	40 (4.0%)
**Scores of Liver disease severity**	
MELD-Na Score, Median (IQR)	10 (8–15)
**UGIB risk stratification Scores**	
GBS, Median (IQR)	7 (3–10)
ABC Score, Median (IQR)	5 (2–7)
MAP(ASH) Score, Median (IQR)	3 (1–4)
Japanese Score, Median (IQR)	1 (0–3)
CHAMPS Score, Median (IQR)	2 (1–3)
**Outcomes**	
Inpatient Death, *n* (%)	94 (9.4%)
Inpatient Complication composite, *n* (%) *	77 (7.7%)
- Rebleeding, *n*	42 (4.2%)
- Repeat OGD/rescoping, *n*	46 (4.6%)
- Interventional radiology, *n*	10 (1.0%)
**Primary cause of Inpatient Death**	
Cause Unrecorded, *n* (%)	39 (41.5%)
Gastrointestinal System, *n* (%)	21 (22.3%)
Respiratory System, *n* (%)	11 (11.7%)
Cardiovascular System, *n* (%)	10 (10.6%)
Renal System, *n* (%)	2 (2.1%)
Neurological System, *n* (%)	1 (1.1%)
Infectious cause, *n* (%)	5 (5.3%)
Oncological cause, *n* (%)	2 (2.1%)
Multi-Organ Failure, *n* (%)	3 (3.2%)

**Abbreviations**: **IQR** = interquartile range; **SD** = standard deviation; **BMI** = Body Mass Index; **ECOG** = Eastern Cooperative Oncology Group; **UGIB** = upper gastrointestinal bleeding; **SBP** = Systolic Blood Pressure; **HR** = Heart Rate; **eGFR** = estimated glomerular filtration rate; **MI** = Myocardial Infarction; **CCF** = congestive Cardiac Failure; **CVA** = Cerebral Vascular Accident; **TIA** = transient ischaemic attack; **HTN** = Hypertension; **COPD** = Chronic Obstructive Pulmonary disease; **ILD** = Interstitial Lung disease; **IDA** = Iron Deficiency Anaemia; **CKD** = chronic kidney disease; **NSAIDs** = non-steroidal anti-inflammatory drugs; **MELD-Na** = Model for End-Stage Liver Disease–sodium;; **GBS** = Glasgow-Blatchford Score; **ABC** = Age–Blood tests–Comorbidities score; **MAP(ASH)** = Mental status–American Society of Anesthesiologists class–Pulse–Albumin–Systolic blood pressure–Haemoglobin score; **CHAMPS** = Charlson Index–Hospital-onset–Albumin–Mental status–Performance–Steroid use; **OGD** = oesophagogastroduodenoscopy. * Components of the inpatient complication composite were not mutually exclusive. Eighteen patients experienced both rebleeding and repeat OGD, one experienced rebleeding and interventional radiology, and two experienced repeat OGD and interventional radiology; no patient experienced all three components.

**Table 2:** Area under the receiver operating characteristic curve (AUROC) with 95% confidence intervals (CIs) for prediction of inpatient mortality and inpatient complications by each risk score overall and within exploratory patient subgroups. Interaction *p*-values were derived from logistic regression models including score-by-subgroup interaction terms.

Score	AUROC (95% CI, *p*-Value)							Effect Modification Across Subgroup (*p*-Values)
	Mortality (Total Cohort)	Complications Composite (Total Cohort)	Non-Variceal Subgroup Mortality	Variceal Subgroup Mortality	Male Subgroup Mortality	Female Subgroup Mortality	Sex (Male vs. Female Subgroups)	Bleeding Cause (Variceal vs. Non-Variceal Subgroups)
**ABC**	0.817 (0.779–0.855, <0.001)	0.651 (0.592–0.709, <0.001)	0.818 (0.777–0.859, <0.001)	0.802 (0.674–0.930, <0.001)	0.805 (0.749–0.862, <0.001)	0.836 (0.786–0.885, <0.001)	0.669	0.341
**CHAMPS**	0.791 (0.745–0.836, <0.001)	0.671 (0.612–0.730, <0.001)	0.799 (0.750–0.848, <0.001)	0.743 (0.619–0.867, <0.001)	0.788 (0.725–0.851, <0.001)	0.793 (0.727–0.859, <0.001)	0.857	0.713
**MAP(ASH)**	0.774 (0.731–0.816, <0.001)	0.697 (0.645–0.748, <0.001)	0.789 (0.742–0.836, <0.001)	0.610 (0.488–0.732, 0.078)	0.767 (0.707–0.826, <0.001)	0.781 (0.718–0.843, <0.001)	0.823	0.099
**GBS**	0.713 (0.660–0.765, <0.001)	0.670 (0.613–0.727, <0.001)	0.724 (0.666–0.782, <0.001)	0.565 (0.417–0.712, 0.381)	0.691 (0.621–0.761, <0.001)	0.743 (0.664–0.821, <0.001)	0.646	0.341
**Japanese**	0.613 (0.553–0.673, <0.001)	0.550 (0.479–0.621, 0.164)	0.603 (0.538–0.669, 0.002)	0.584 (0.447–0.720, 0.070)	0.649 (0.567–0.732, <0.001)	0.577 (0.491–0.663, 0.081)	0.251	0.765

## Data Availability

Data are available from the corresponding author upon reasonable request, subject to institutional data governance.

## Supplementary Materials

The following supporting information can be downloaded at: https://www.mdpi.com/article/10.3390/jcm15197503/s1. Supplementary Methods. Data Extraction and Variables Collected. Figure S1. ROC curves showing the predictive performance of the respective risk scoring systems for inpatient mortality, stratified by subgroup: (A) NVUGIB subgroup; (B) variceal subgroup; (C) male subgroup; and (D) female subgroup. Figure S2. Comparison of AUROCs of the five UGIB risk scores for all-cause inpatient mortality across bleeding-aetiology and sex subgroups. Figure S3. Receiver operating characteristic curves for prediction of all-cause inpatient mortality using (A) ABC alone and (B) a model incorporating ABC and BMI. Table S1. Components, definitions and data sources used for retrospective reconstruction of the five UGIB prognostic scores. The following scores were calculated at admission based on original published criteria. Score ranges: GBS, 0–23; ABC, 0–18; MAP(ASH), 0–9; Japanese score, −4 to +10; CHAMPS, 0–7 [3,4,5,6,7]. Laboratory variables were obtained from the first blood tests on admission and physiological variables from the first recorded observations on admission. Clinical variables and comorbidities were derived from the electronic health record. ASA physical status, ECOG performance status, and altered mental status were reconstructed from documented clinical information. Table S2. Missing data for variables included in the study analyses (*n* = 1003). Missingness is reported relative to the full cohort (*n* = 1003). Analyses were performed using available cases according to variable availability. Table S3. Baseline characteristics of patients with UGIB, stratified by variceal vs. non-variceal bleeding. Table S4. Pairwise comparison of AUROCs using DeLong’s test. ΔAUROC represents AUROC of Score 1 minus AUROC of Score 2. Pairwise comparisons were performed using DeLong’s test. Positive ΔAUROC values indicate higher discrimination for Score 1, whereas negative values indicate higher discrimination for Score 2. A two-sided *p*-value <0.05 was considered statistically significant. Table S5. Diagnostic performance of evaluated low-risk thresholds for inpatient mortality. Low-risk classification was based on the threshold shown for each score. Sensitivity, specificity, PPV, and NPV are reported with 95% confidence intervals. Denominators vary slightly because of missing score data. Table S6. Comparison of ABC and MELD-Na for predicting all-cause inpatient mortality among patients with variceal upper gastrointestinal bleeding. Analysis was restricted to patients with variceal upper gastrointestinal bleeding with complete data for both ABC and MELD-Na (*n* = 106; 15 inpatient deaths). *ΔAUROC represents ABC minus MELD-Na; the AUROCs were compared using DeLong’s test for correlated ROC curves. Table S7. Liver disease severity and coagulation parameters according to inpatient survival among patients with documented cirrhosis. Table S8. Association between BMI category and all-cause inpatient mortality after adjustment for ABC score among patients with recorded BMI (*n* = 748). Underweight: BMI < 18.5; Healthy (reference group): BMI 18.5–24.9; Overweight: BMI 25.0–29.9; Obese: BMI 30.0–39.9; Severely obese: BMI ≥ 40.0. Table S9. Comparison of patients with and without recorded BMI category. Patients were classified according to whether BMI category could be determined from the available data. Comparisons were performed using available cases. Table S10. Comparison of discrimination for all-cause inpatient mortality between ABC alone and ABC with BMI incorporated.
