# Supplementary material for: Validation and Performance of Five Upper Gastrointestinal Bleeding Prognostication Scores in a Single-Centre United Kingdom Cohort of over 1000 Patients
Source: J Clin Med. 2026 Sep 27;15(19):7503. doi: 10.3390/jcm15197503 (PMC13666153; doi:10.3390/jcm15197503)
Supplement: Supplementary file 1 [file jcm-15-07503-s001.zip › jcm-4525269-supplementary.pdf]

Title: Validation and performance of 5 upper gastrointestinal bleeding prognostication scores in a United Kingdom cohort of over 1000 patients.

Short title: Comparative performance of prognostic scores for UGIB.

Authors: Mohamed Jalloh[1], Balamrit Singh Sokhal[1,2], Maryam Javed[1], Humberto Gomez[1], Jessica Lightfoot[1], Farhan Gohar[1*]

1. Royal Stoke University Hospital, University Hospitals North Midlands, Stoke-On-Trent, Staffordshire, United Kingdom
2. School of Medicine, Keele University, Keele, Staffordshire, United Kingdom

Supplementary Methods

Data Extraction and Variables Collected

Clinical data were retrospectively extracted from electronic medical records. The following variables were collected:

- **Presenting symptoms**: melaena, haematemesis, and coffee-ground vomit.
- **Clinical signs**: encephalopathy, and ascites.
- **Vital signs**: systolic and diastolic blood pressure, and heart rate.
- **Performance status**: Eastern Cooperative Oncology Group Performance status (ECOG-PS).
- **Anaesthesiology classification**: American Society of Anaesthesiologists (ASA) score.
- **Laboratory values**: sodium, urea, creatinine, eGFR, albumin, bilirubin, haemoglobin, platelets, and INR.
- **Medication history**: antiplatelet, anticoagulant, steroid, and non-steroidal anti-inflammatory drugs (NSAIDs) use.
- **Comorbidities**: myocardial infarction, heart failure, peripheral vascular disease, cerebrovascular accident, diabetes, hypertension, chronic lung disease, chronic kidney disease stage 4-5 (CKD stage 4-5), liver cirrhosis, and active malignancy.
- **Demographics**: age, sex, ethnicity, and BMI.

Score Definitions

The following scores were calculated at admission based on original published criteria. [3-7]

Glasgow-Blatchford Score (GBS)

- Blood urea nitrogen (mg/dL)
- Gender
- Haemoglobin (g/L)
- Systolic BP (mmHg)
- Heart rate (bpm)
- Melena
- Syncope
- Hepatic disease
- Cardiac failure

Total score: 0-23.

ABC

- Age
- Urea (mmol/L)
- Albumin (g/L)
- Creatinine (µmol/L)
- Altered mental status
- Liver cirrhosis
- Disseminated malignancy
- ASA score

Total score: 0–18.

MAP(ASH) Score

- Mental status altered
- ASA score
- Heart rate (bpm)
- Albumin (g/L)
- Systolic (mmHg)
- Haemoglobin (g/L)

Total score: 0–9.

Japanese Score

- Systolic Blood pressure (mmHg)
- Syncope
- Hematemesis
- Haemoglobin (g/L)
- Blood urea nitrogen (mg/dL)
- eGFR
- Antiplatelet use

Total score: -4 - +10.

CHAMPS Score

- Charlson Comorbidity Index (CCI)
- Hospital-onset bleeding
- Albumin (g/L)
- Altered mental status
- ECOG Performance Status
- Steroid use

Total score: 0–7.

Charlson Comorbidity Index (CCI) Score

- Myocardial infarction
- Congestive heart failure
- Peripheral vascular disease
- Cerebrovascular disease
- Dementia
- Chronic pulmonary disease
- Connective tissue disease
- Peptic ulcer disease
- Mild liver disease
- Diabetes without complications
- Diabetes with complications
- Hemiplegia
- Moderate or severe kidney disease
- Solid localised Malignant tumour
- Leukaemia
- Lymphoma
- Moderate or severe liver disease
- Metastatic solid tumour
- AIDS

Supplementary Figures

Supplementary Figure S1. ROC curves showing the predictive performance of the respective risk scoring systems for inpatient mortality, stratified by subgroup: **(A)** NVUGIB subgroup; **(B)** variceal subgroup; **(C)** male subgroup; and **(D)** female subgroup.

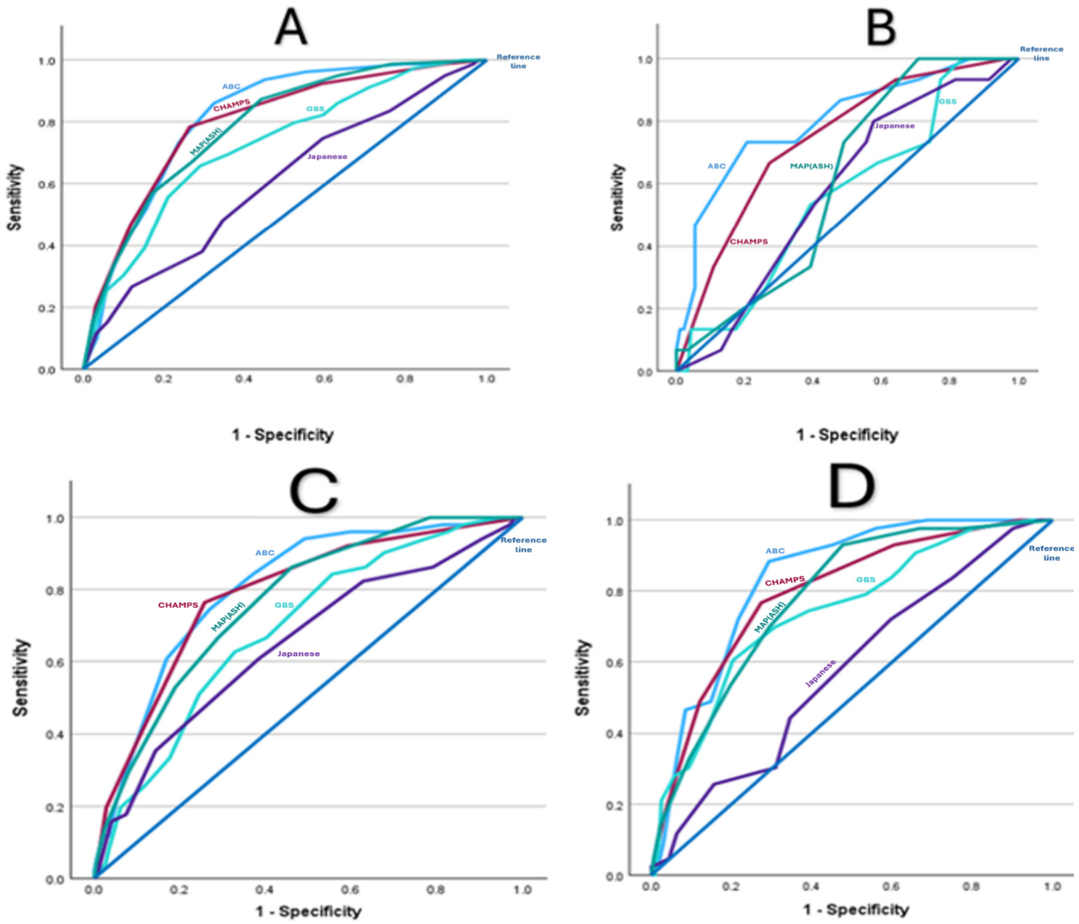

Supplementary Figure S2. Comparison of AUROCs of the five UGIB risk scores for all-cause inpatient mortality across bleeding-aetiology and sex subgroups.

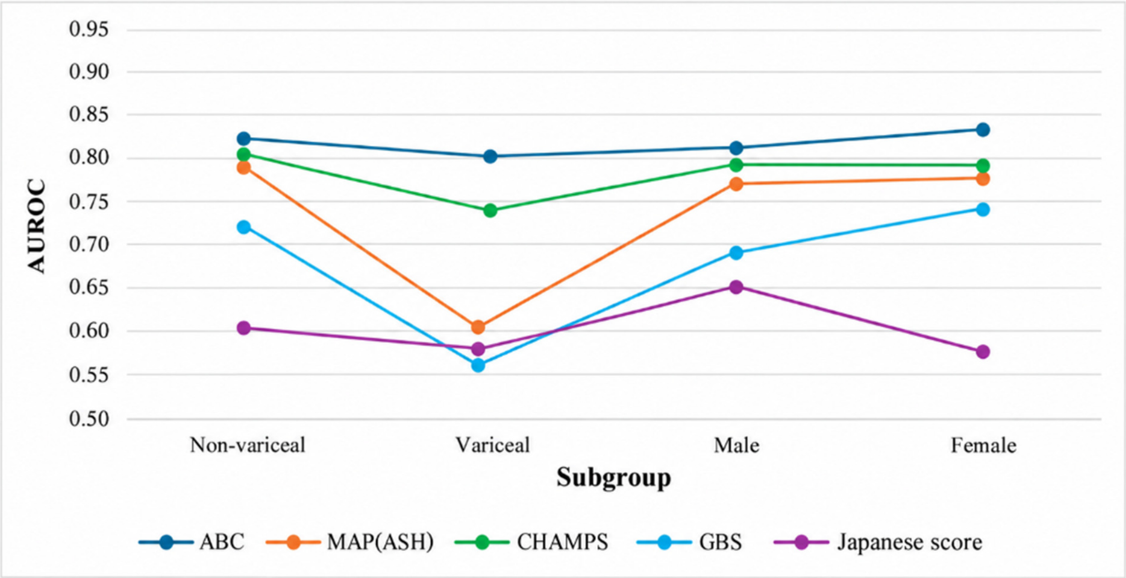

Supplementary Figure S3. Receiver operating characteristic curves for prediction of all-cause inpatient mortality using **(A)** ABC alone and **(B)** a model incorporating ABC and BMI.

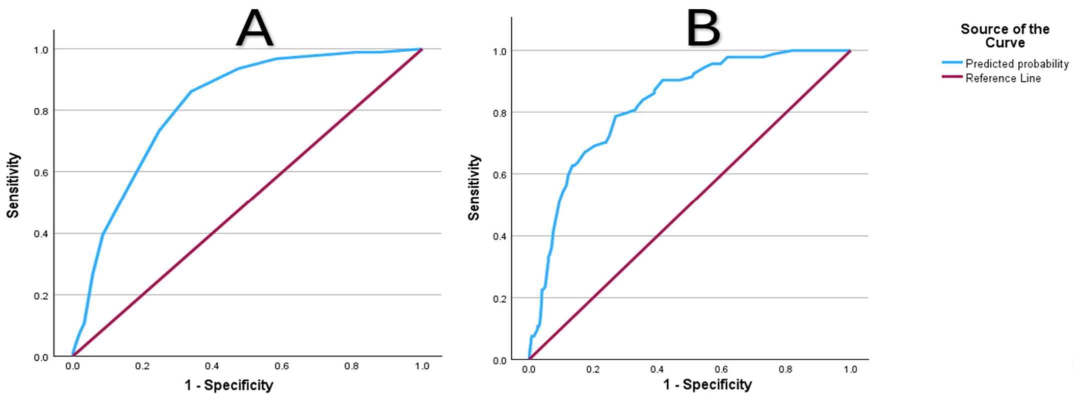

Supplementary Tables

Supplementary Table S1. Components, definitions and data sources used for retrospective reconstruction of the five UGIB prognostic scores. The following scores were calculated at admission based on original published criteria. **Score ranges:** GBS, 0–23; ABC, 0–18; MAP(ASH), 0–9; Japanese score, −4 to +10; CHAMPS, 0–7. [3-7] Laboratory variables were obtained from the first blood tests on admission and physiological variables from the first recorded observations on admission. Clinical variables and comorbidities were derived from the electronic health record. ASA physical status, ECOG performance status and altered mental status were reconstructed from documented clinical information.

**Score**	**Component**	**Definition / scoring approach**	**Retrospective data source**
**GBS**	Blood urea nitrogen	Scored according to original published GBS criteria	First blood tests obtained on admission
	Sex	Scored according to original published criteria	Demographic record
	Haemoglobin	Sex-specific scoring according to original published criteria	First blood tests obtained on admission
	Systolic blood pressure	Scored according to original published criteria	First recorded observations on admission

	Heart rate	Scored according to original published criteria	First recorded observations on admission
	Melaena	Presence/absence	Presenting symptoms documented in the electronic health record
	Syncope	Presence/absence	Presenting symptoms documented in the electronic health record
	Hepatic disease	Presence/absence	Documented medical history
	Cardiac failure	Presence/absence	Documented medical history
**ABC**	Age	Scored according to original published ABC criteria	Demographic record
	Urea	Scored according to original published criteria	First blood tests obtained on admission

	Albumin	Scored according to original published criteria	First blood tests obtained on admission
	Creatinine	Scored according to original published criteria	First blood tests obtained on admission
	Altered mental status	Presence/absence according to original criteria	Clinical documentation in the electronic health record
	Liver cirrhosis	Presence/absence	Documented medical history
	Disseminated malignancy	Presence/absence	Documented medical history
	ASA score	Scored according to original published criteria	Determined retrospectively from documented clinical information
**MAP(ASH)**	Altered mental status	Scored according to original MAP(ASH) criteria	Clinical documentation in the electronic health record
	ASA score	Scored according to original published criteria	Determined retrospectively from

			documented clinical information
	Heart rate	Scored according to original published criteria	First recorded observations on admission
	Albumin	Scored according to original published criteria	First blood tests obtained on admission
	Systolic blood pressure	Scored according to original published criteria	First recorded observations on admission
	Haemoglobin	Scored according to original published criteria	First blood tests obtained on admission
**Japanese score**	Systolic blood pressure	Scored according to original published Japanese-score criteria	First recorded observations on admission
	Syncope	Presence/absence	Presenting symptoms documented in the electronic health record
	Haematemesis	Presence/absence	Presenting symptoms

			documented in the electronic health record
	Haemoglobin	Scored according to original published criteria	First blood tests obtained on admission
	Blood urea nitrogen	Scored according to original published criteria	First blood tests obtained on admission
	eGFR	Scored according to original published criteria	First blood tests obtained on admission
	Antiplatelet use	Presence/absence	Medication history documented in the electronic health record
**CHAMPS**	Charlson Comorbidity Index	Calculated from documented comorbidities according to the original CCI weighting system	Medical history documented in the electronic health record
	Hospital-onset bleeding	Presence/absence	Clinical documentation in the

			electronic health record
	Albumin	Scored according to original published CHAMPS criteria	First blood tests obtained on admission
	Altered mental status	Presence/absence according to original criteria	Clinical documentation in the electronic health record
	ECOG performance status	Scored according to original published criteria	Determined retrospectively from documented clinical information
	Steroid use	Presence/absence according to original criteria	Medication history documented in the electronic health record

Supplementary Table S2. Missing data for variables included in the study analyses (n = 1,003). Missingness is reported relative to the full cohort (n = 1,003). Analyses were performed using available cases according to variable availability.

**Variable**	**Missing, n (%)**
BMI category	255 (25.4%)
MELD-Na score	75 (7.5%)
Platelet count	27 (2.7%)
Bilirubin	9 (0.9%)
Diabetes status	6 (0.6%)
Albumin	4 (0.4%)
MAP(ASH) score	4 (0.4%)
CHAMPS score	4 (0.4%)
eGFR	2 (0.2%)
ABC score	1 (0.1%)
GBS score	1 (0.1%)
Japanese score	1 (0.1%)

Supplementary Table S3. Baseline characteristics of patients with UGIB, stratified by variceal vs non-variceal bleeding.

Variable	Non-variceal (n = 896)	Variceal (n = 107)	p-value
**Demographics**			
Male sex, n (%)	539 (60.2%)	55 (51.4%)	0.082
Age, years	73 (60–82)	61 (55–67)	<0.001
BMI, kg/m²	26.0 (23–30)	26.7 (23–30)	0.766
Ethnicity, n (%)			0.865
Caucasian	821 (91.6%)	100 (93.5%)	
African	8 (0.9%)	0 (0.0%)	
Asian	15 (1.7%)	2 (1.9%)	
Mixed	12 (1.3%)	0 (0.0%)	
Not recorded	40 (4.5%)	5 (4.7%)	
**Vital signs on admission**			
Systolic blood pressure, mmHg	125 (112–141)	122 (105–135)	0.015
Heart rate, beats/min	85 (75–97)	96 (82–107)	<0.001
**Laboratory blood tests**			
Sodium, mmol/L	139 (136–141)	138 (134–140)	0.001
Urea, mmol/L	9.0 (5.6–14.7)	7.7 (3.9–12.7)	0.021
Creatinine, µmol/L	82 (62–118)	66.5 (51–91)	<0.001
eGFR, mL/min/1.73 m²	78 (47–90)	90 (67–90)	<0.001

Haemoglobin, g/L	96 (80–119)	86 (78–100.5)	<0.001
Albumin, g/L	28.6 ± 7.9	24.7 ± 5.4	<0.001
**Comorbidities**			
Myocardial infarction, n (%)	113 (12.6%)	6 (5.6%)	0.034
Congestive cardiac failure, n (%)	113 (12.6%)	3 (2.8%)	0.003
Cerebrovascular accident, n (%)	106 (11.8%)	5 (4.7%)	0.026
Hypertension, n (%)	446 (49.8%)	26 (24.3%)	<0.001
Iron-deficiency anaemia, n (%)	77 (8.6%)	6 (5.6%)	0.289
Dementia, n (%)	45 (5.0%)	1 (0.9%)	0.056
Chronic lung disease (COPD/asthma/ILD), n (%)	198 (22.1%)	24 (22.4%)	0.938
Decompensated cirrhosis, n (%)	70 (7.8%)	82 (76.6%)	<0.001
Diabetes requiring ≥1 medication, n (%)	176 (19.8%)*	30 (28.0%)	0.057
CKD stage 4–5, n (%)	61 (6.8%)	1 (0.9%)	0.017
Active cancer (including metastatic disease), n (%)	192 (21.4%)	8 (7.5%)	0.001

**Liver disease severity**			
MELD-Na score	9.5 (8–14)	16 (12–20.75)	<0.001
**UGIB risk scores**			
Glasgow-Blatchford score	7 (3–10)	9 (7–11)	<0.001
ABC score	4 (2–7)	6 (4–8)	<0.001
MAP(ASH) score	2 (1–4)	4 (2.5–5)	<0.001
Japanese score	1 (0–3)	3 (1–4)	<0.001
CHAMPS score	2 (1–3)	2 (1–3)	0.244
**Outcomes**			
Inpatient complication composite, n (%)	60 (6.7%)	17 (15.9%)	<0.001
All-cause inpatient mortality, n (%)	79 (8.8%)	15 (14.0%)	0.081

*Diabetes status was unavailable for six patients in the non-variceal group; percentages are based on available cases.

Supplementary Table S4. Pairwise comparison of AUROCs using DeLong's test. ΔAUROC represents AUROC of Score 1 minus AUROC of Score 2. Pairwise comparisons were performed using DeLong's test. Positive ΔAUROC values indicate higher discrimination for Score 1, whereas negative values indicate higher discrimination for Score 2. A two-sided p-value <0.05 was considered statistically significant.

**Outcome**	**Score 1**	**AUROC 1**	**Score 2**	**AUROC 2**	**ΔAUROC (95% CI for ΔAUROC, p-value)**
**Inpatient mortality**	ABC	0.817	CHAMPS	0.791	0.026 (−0.015 to 0.067, 0.221)
	ABC	0.817	MAP(ASH)	0.774	0.043 (−0.004 to 0.090, 0.070)
	ABC	0.817	GBS	0.713	0.105 (0.048 to 0.161, <0.001)
	ABC	0.817	Japanese	0.613	0.204 (0.132 to 0.276, <0.001)
	CHAMPS	0.791	MAP(ASH)	0.774	0.017 (−0.025 to 0.059, 0.421)
	CHAMPS	0.791	GBS	0.713	0.078 (0.017 to 0.139, 0.012)
	CHAMPS	0.791	Japanese	0.613	0.177 (0.103 to 0.251, <0.001)
	MAP(ASH)	0.774	GBS	0.713	0.061 (0.018 to 0.104, 0.005)
	MAP(ASH)	0.774	Japanese	0.613	0.160 (0.098 to 0.222, <0.001)
	GBS	0.713	Japanese	0.613	0.099 (0.039 to 0.160, 0.001)

**Inpatient complication composite**	ABC	0.651	CHAMPS	0.671	−0.020 (−0.074 to 0.032, 0.436)
	ABC	0.651	MAP(ASH)	0.697	−0.046 (−0.101 to 0.007, 0.090)
	ABC	0.651	GBS	0.670	−0.019 (−0.078 to 0.040, 0.531)
	ABC	0.651	Japanese	0.550	0.101 (0.010 to 0.192, 0.030)
	CHAMPS	0.671	MAP(ASH)	0.697	−0.026 (−0.077 to 0.025, 0.324)
	CHAMPS	0.671	GBS	0.670	0.001 (−0.066 to 0.069, 0.969)
	CHAMPS	0.671	Japanese	0.550	0.121 (0.031 to 0.210, 0.008)
	MAP(ASH)	0.697	GBS	0.670	0.027 (−0.013 to 0.067, 0.188)
	MAP(ASH)	0.697	Japanese	0.550	0.147 (0.076 to 0.217, <0.001)
	GBS	0.670	Japanese	0.550	0.120 (0.051 to 0.189, <0.001)

Supplementary Table S5. Diagnostic performance of evaluated low-risk thresholds for inpatient mortality. Low-risk classification was based on the threshold shown for each score. Sensitivity, specificity, PPV and NPV are reported with 95% confidence intervals. Denominators vary slightly because of missing score data.

Risk score	Low-risk threshold	Patients classified as low risk, n/N (%)	Sensitivity, % (95% CI)	Specificity, % (95% CI)	PPV, % (95% CI)	NPV, % (95% CI)
ABC	≤3	383/1002 (38.2%)	96.8 (91.0–99.3)	41.9 (38.6–45.1)	14.7 (12.0–17.7)	99.2 (97.7–99.8)
CHAMPS	=0	91/999 (9.1%)	98.9 (94.2–100.0)	9.9 (8.1–12.1)	10.2 (8.3–12.4)	98.9 (94.0–100.0)
MAP(ASH)	≤2	492/999 (49.2%)	89.4 (81.3–94.8)	53.3 (49.9–56.6)	16.6 (13.4–20.1)	98.0 (96.3–99.0)
GBS	≤1	150/1002 (15.0%)	97.9 (92.5–99.7)	16.3 (14.0–18.9)	10.8 (8.8–13.1)	98.7 (95.3–99.8)
Japanese score	≤0	369/1002 (36.8%)	77.7 (67.9–85.6)	38.3 (35.2–41.6)	11.5 (9.1–14.3)	94.3 (91.4–96.4)

Supplementary Table S6. Comparison of ABC and MELD-Na for predicting all-cause inpatient mortality among patients with variceal upper gastrointestinal bleeding. Analysis was restricted to patients with variceal upper gastrointestinal bleeding with complete data for both ABC and MELD-Na (n = 106; 15 inpatient deaths). *ΔAUROC represents ABC minus MELD-Na; the AUROCs were compared using DeLong's test for correlated ROC curves.

Score	N	Inpatient deaths, n	AUROC (95% CI)	ΔAUROC (95% CI)*	p-value*
**ABC**	106	15	0.800 (0.672–0.929)	0.199 (0.002–0.396)	0.048
**MELD-Na**	106	15	0.601 (0.442–0.761)	—	—

Supplementary Table S7. Liver disease severity and coagulation parameters according to inpatient survival among patients with documented cirrhosis.

**Characteristic**	**Survived (n = 149)**	**Died (n = 29)**	**p-value**
INR, median (IQR)	1.50 (1.30–1.87)	1.65 (1.58–1.90)	0.057
Platelet count, $\times 10^9$/L, median (IQR)	155 (86–205)	124 (84–172)	0.307
MELD-Na score, median (IQR)	15 (11.2–20)	21 (15.8–23)	0.002

Supplementary Table S8. Association between BMI category and all-cause inpatient mortality after adjustment for ABC score among patients with recorded BMI (n = 748)

Underweight: BMI < 18.5; Healthy (reference group): BMI 18.5–24.9; Overweight: BMI 25.0–29.9; Obese: BMI 30.0–39.9; Severely obese: BMI ≥ 40.0

**Variable**	**Adjusted OR (95% CI)**	**p-value**
ABC score	1.459 (1.327–1.616)	<0.001
BMI category		0.702*
Healthy weight	Reference	—
Underweight	1.116 (0.295–3.397)	0.857
Overweight	0.855 (0.423–1.728)	0.660
Obese	1.514 (0.697–3.249)	0.288
Severely obese	0.972 (0.126–4.708)	0.975

Supplementary Table S9. Comparison of patients with and without recorded BMI category. Patients were classified according to whether BMI category could be determined from the available data. Comparisons were performed using available cases.

Characteristic	BMI recorded (n = 748)	BMI not recorded (n = 255)	p-value
Age, years, mean ± SD	68.2 ± 16.1	62.1 ± 20.4	<0.001
Male sex, n (%)	447 (59.8%)	147 (57.6%)	0.556
ABC score, mean ± SD	4.87 ± 3.04	4.25 ± 3.35	0.010
Variceal bleeding, n (%)	77 (10.3%)	30 (11.8%)	0.557
All-cause inpatient mortality, n (%)	59 (7.9%)	35 (13.7%)	0.009

Supplementary Table S10. Comparison of discrimination for all-cause inpatient mortality between ABC alone and ABC with BMI incorporated.

**Model**	**AUROC**	**Pairwise comparison**	**p-value**
ABC score alone	0.817	Reference	—
ABC score + BMI	0.828	ABC + BMI vs ABC alone	0.224
